# G3DMS: Design and Implementation of a Data Management System for the Diagnosis of Genetic Disorders

**DOI:** 10.3390/healthcare8030196

**Published:** 2020-07-03

**Authors:** Halima Samra, Alice Li, Ben Soh

**Affiliations:** 1 Department of Comp. Science and Information Technology, La Trobe University, Victoria 3086, Australia; A.Li@latrobe.edu.au (A.L.); B.Soh@latrobe.edu.au (B.S.); 2 Faculty of Computing and Information Technology, King Abdulaziz University, Jeddah 21589, Saudi Arabia

**Keywords:** data management system, clinical research databases, Barker methodology, medical research data, genetic diagnosis data, genetic testing data, clinical information systems, Saudi Arabia

## Abstract

Current health information systems used in genetic research centers and clinics in the Kingdom of Saudi Arabia have failed to enable researchers and health care physicians to utilize genetic and clinical data in their research. In this paper, we aim to design and implement a Genetic Disorders Diagnosis Data Management System (G3DMS) to support clinicians in the process of diagnosing genetic diseases and conducting genetic studies. A case study was undertaken to analyze a health information system in Saudi to understand its design problems via a brainstorming method. We then used the Barker’s system design method and a prototype to validate our proposed system via usability testing. This research has resulted in the development of the G3DMS that comprises: electronic data-capture forms for data entry; a customized query builder to display and modify patient data as well as form research queries; a module that allows historical data to be uploaded in the form of bulk data using a template; export data options to Excel and JavaScript Object Notation (JSON) format; and authorization access for healthcare researchers and clinicians. The G3DMS was implemented in the Princess Al-Jawhara Center of Excellence in Research of Hereditary Disorders, Jeddah, KSA.

## 1. Introduction

A health information system (HIS) is used in healthcare to facilitate the clinical care process by capturing, storing, processing and delivering information to decision-makers. A HIS is an important component of hospital information system solutions, which include the electronic medical records (EMR) system, computerized physician order entry (CPOE) system, laboratory information system (LIS), pharmacy information system (PIS), and radiology information system (RIS) [1], as well as the emerging electronic health record (EHR) in care settings which comprise a digitized version of all clinical data relevant to the patients’ care such as demographics, medical history, medication, care plan, laboratory data, radiology reports, physicians’ feedback, and billing information [2]. The proper application of HIS has the potential to improve healthcare delivery and enable decision-makers to take informed action based on integrated data. The successful development and implementation of HIS can improve the efficiency and effectiveness of healthcare services and outcomes [3]. However, barriers to the implementation of HIS results in adoption variation among organizations or even countries around the world [4]. The adoption rate is affected by different factors that influence the implementation outcome of HIS, which can be categorized as ethical, financial, functionality, organizational, political, technical, and training [5].

Saudi Arabia is one of the developing countries that over the past few years has achieved significant progress in broadening the adoption of health information technology. The application of HIS in Saudi hospitals varies, as some hospitals lack computerized systems, and other hospitals are using HIS from different vendors [6]. For example, the adoption rate of EHRs and EMRs in Saudi Arabia is growing at differential adoption levels according to the hospital size and type and ownership throughout all regions with multiple challenges that delay their full implementation [7]. Despite the perceived effort from the government to encourage health organizations to accelerate the adoption process of HIS, barriers arise as they approach system implementation [7]. Technical, administrative, and financial factors are identified as critical barriers to the successful implementation, as well as human barriers [8]. Difficulties in HIS implementation result in many issues within the Saudi health system and affect healthcare professionals in their daily workflow as well as their research contribution due to a lack of quality data. In our previous work, we conducted a mixed-method study to examine and analyze the current state of clinical and genetic data available for medical research in Saudi hospitals, clinics, and research centers [9]. The focus was on identifying the major issues that hindered the exploitation of healthcare data sources such as EMR or EHR for medical research. Accordingly, we intend to highlight possible solutions to enhance the role of HIS in medical research in the Kingdom of Saudi Arabia. Data collection, data storage, and a lack of system interoperability were the major impediments facing healthcare professionals in managing the data required for their research [9]. The implementation of EMR and EHR is limited to major large-sized hospitals, as small-sized hospitals and clinics do not have adequate computerized systems for routine data collection and management [6]. Therefore, physicians in the Kingdom of Saudi Arabia, especially in critical areas such as genetic clinics and research centers, face great challenges with the current methods of data collection and storage (Excel files) which makes identifying patient data for follow-up and research a difficult task.

Motivated by the initiative to contribute to the field of health information management, we focus on the area of genetic data provision for medical research in Saudi Arabia for the following reasons:

Saudi Arabia has one of the highest consanguinity rates in the world as first cousin marriages constitute 60–70% of all marriages [10]. This has resulted in the prevalence of rare inherited genetic disorders that have become quite common in Saudi Arabia, nearly double the rate in Europe and the U.S. and ten times higher for specific diseases.

Although the government supports many projects and programs that aim to collect genetic data such as the Saudi Human Genome Program to help research and develop preventative measures to limit the prevalence of diseases in the region [11], the lack of uniformity and data silos due to individual efforts to obtain clinical and genetic data through centers and clinics has made it more difficult for researchers to pre-process and manage data associated with their studies [12].

Genetic clinics and research centers have helped to reveal genetic disorders and generate curated data which can be a valuable source for researchers if the data are collected properly and stored efficiently. Sharing such data could advance research and enhance healthcare quality by providing accurate information on commonly encountered inherited disorders and lower the incidence of genetic diseases using preventive measures such as premarital and preimplantation testing [12]. However, these centers lack adequate systems for managing and archiving patient data for research, making it difficult to integrate and build a national genetic diseases database.

Internal problems within Saudi clinics and genetic research centers in terms of data collection and retention for use in research hinders data integration and sharing for research and the establishment of a unified database of genetic diseases [13].

Previous studies in the area of HIS globally and within the Saudi context have focused on the implementation, effectiveness, and impact of HIS in identifying the adoption barriers and identifying successful models from other nations as well as presenting recommendations to overcome these obstacles. However, these studies are not able to present a solution to solve the data management issue in healthcare settings, and they lack technical models to solve HIS issues such as integration mechanisms for new HIS fusion to legacy systems. As far as we know, none of the existing papers describes the development and application of a novel system which can serve a dual role in terms of daily patient care and research use specifically in the Saudi context. Therefore, this paper attempts to bridge this gap.

This paper details the design and implementation processes of a novel system, the Genetic Disorders Diagnostic Data Management System (G3DMS). In order to achieve the intended system, this paper first investigates the theoretical framework for the design of a health information management system and identifies the system components and examines some frameworks to evaluate the implementation of the system during the system design lifecycle. We then use a case study to analyze a health information system in Saudi to understand its design problems. The Barker’s system design method is used to organize the development process in seven fundamental steps, taking into consideration health informatics frameworks and employing evaluation models of influencing factors on successful HIS implementation and adoption of a new system in the healthcare environment.

## 2. Theoretical Considerations

The design and implementation of HIS require a consideration of the underlying theoretical frameworks of such a system, which includes the system (software) development lifecycle from a computer science perspective and a health informatics framework to evaluate the success and failure of HIS implementation and its adoption rate. This paper adopts methods and frameworks from both fields to provide a comprehensive design for the G3DMS as well as to ensure successful implementation and guarantee the satisfaction of the system users for full adoption.

### 2.1. The Design of a Data Management System

The structure of the data management system consists of two fundamental parts, the database which constitutes the back-end of the system and the application interface, the system front-end. Database design focuses on the use of the database architecture to store and manage data for the end-user [14]. The principal purpose is to create reliable and useful data models that can effectively function as tools for communicating with potential system users and as blueprints for database developers. In order for the design process to produce a high-quality product for the customer, the basic steps of the database design process must be clear and provide a well-organized step-by-step guide to database design [15]. Before implementing design efforts, developers must understand the business processes and information requirements (rules, entities, and attributes), and then convert the business attributes into a business model, which will later contribute to transforming the resulting business model into a database model using the design methodology [16]. The lifecycle approach addresses all stages from start to finish in an orderly and systematic manner. The process of the database lifecycle starts by selecting a relevant approach (a data-driven approach, a process-driven approach, or a parallel/blended approach) using a set of business rules, processes, and data that have been defined [15]. The development process involves several steps depending on the methodology followed, for instance, the traditional design method, the Barker method, or other adapted design methods [17]. The traditional method includes three phases: business requirements analysis, data modelling, and the normalization phase [15]. Barker method: named after Richard Barker, who was responsible for designing the Oracle Automatic Design Tool (referred to as the CASE Tool). The Barker method exceeded the traditional method by providing more steps that better organize the design effort. The Barker method comprises seven phases: Strategy, Analysis, Design, Build, Documentation, Transition, and Production. In the strategy phase, business needs are addressed using a strategy document to guide the next phase using a strategy entity-relationship diagram (ERD) to break down the basic categories of the business entities and the process flow diagrams to show the basic flow of parent-child processes. At the analysis phase, all requirements should be gathered, and the analysis of the ERD should be used to represent the detailed entities and attribute relationships, primary keys, and constraints. The design phase role is to physically design the schema that will be built based on the strategy and analysis phases. During this phase, the logical process flow diagrams are converted to the physical process flow diagrams and logical ERDs are transformed into diagrams that represent a physical database. The build phase involves the creation of the actual database that has been designed in the physical database system. During this phase, tables and indexes should be designed, hardware components should be utilized, and database environments should be considered. Documentation should be devised for both the information system and the end-user. System documentation targets developers, programmers, database administrators, and technical management, and end-user documentation targets the system user. During the transition phase, precautionary steps should be taken, such as product testing before production and introducing the complete back-end database and the database application interface. The final production phase is where the database is implemented into the production environment and is made available to the end-user for daily use [15].

Regardless of the design methodology chosen, the design process essentially involves the same process of creating a logical model and transforming it into a functioning physical model. The database lifecycle shows what steps are required in the methodical approach to database design and includes logical database design, which is the process of modelling the conceptual level information using a specific data model representation [18].

### 2.2. The Implementation of Successful Health Information System Models

During the implementation of the health information system, different organizational, technical, and human barriers may arise at different levels of the implementation process [19]. Therefore, planning the implementation of any HIS in a healthcare environment, regardless of the resource setting (low-resource or high-resource), requires careful attention to be paid to the critical factors to guarantee successful implementation and a high adoption rate. System quality, ease of use, responsiveness and security are essential factors influencing system implementation in a low-resource setting [5]. However, in a high-resource setting, additional regulatory or environmental factors influence the quality of the system and its use, such as leadership support for system design, development, deployment, and ongoing support for user training [20]. Therefore, to investigate the successful implementation of HIS and to measure the adoption rate, evaluation methods were developed or adopted in many studies based on frameworks structured around factors which positively influence system implementation and adoption. The Information System Success Model is used to define the effects of technological barriers on the user and determine the successful application of the information system based on three criteria: information quality, system quality, and service quality [8]. However, the model focusses only on information technology (IT) and system quality, and it is the only factor that determines the overall effect [21]. The technology acceptance model (TAM) is also used to analyze user acceptance or rejection of a system based on two aspects: “perceived ease of use” and “perceived usefulness” to predict the behavioural intention of the user towards system use [21]. Other models based on the idea of compatibility or “fitness” focus on the clinical workflow and the task required to be accomplished by the user using IT, such as the task-technology-fit model (TTF) which addresses only the fitness between the user and technology and between the task and technology, the FITT framework (“Fit between Individuals, Task and Technology”) which is based on the fit between the attributes of the users, technology, and clinical tasks, and the Human–Organisation–Technology Fit (HOT-fit) model which is based on addressing the socio-technical components of IS and the fit between them throughout the system development lifecycle [19].

### 2.3. Evaluation Frameworks

Although a few studies have been conducted on the adoption of the health information system in developing countries, adopting models to assess the success and failure of the implementation of HIS highlighted real-world applications using case studies in developing countries which resulted in confirming the identified factors or defining new influencing barriers. Bawack and Kamdjoug used a modified model of the technology acceptance model, the unified theory of acceptance and use of technology (UTAUT), to examine the behavioural intention of clinicians in Cameroon to accept and use HIS. The authors confirmed the original UTAUT was inadequate to describe the intention of the clinicians in developing countries, so they extended the model to fit the context. It was found that the model performs better with age as a single moderating factor [22]. Sustainability frameworks which are based on sustainability theories from the literature focus on factors that influence the long-term usage of the system. Moucheraud et al. developed a conceptual framework to explore the potential sustainability of electronic health information systems investment in Malawi, Zambia, and Zimbabwe, as well as to identify the factors likely to contribute to the continuation of donor-supported initiatives after the original support has been modified or ended. Their findings suggest that although maintaining sustainability in a low-resource setting requires intensive efforts, long-term success will be reflected in the improvement of healthcare outcomes [23]. Afrizala et al. developed a research framework to evaluate the implementation of the Primary Health Care Information System in a rural area in Indonesia. Their framework is based on two models, the HOT-fit model (which investigates the factors that influence management’s decision) and the FITT model (which analyses socio-organizational factors that affect IT adoption in healthcare settings). Their results suggest that greater interaction between human resources, infrastructure, organizational support, and process factors is critical to effective adoption [24]. Multicriteria user satisfaction analysis is used to measure user satisfaction and determine the strong and weak points of user satisfaction. Kitsios et al. implemented the MUSA (MUlticriteria Satisfaction Analysis) method to measure the user’s satisfaction of an e-appointment system of a Greek state hospital in Thessaloniki and obtain data to support decision-makers. Their study demonstrated that ease of use and system quality were of secondary importance to patients, although other studies delivered contradictory findings [25]. On the other hand, for successful implementation, public health information systems need to be accommodated within the informatics framework model that supports health information exchange between the healthcare community. The public health informatics framework developed by Gotham et al. suits the dual-use nature of information systems; therefore, it is used to depict all phases of public health emergency preparedness. Their findings emphasize the significant role of a well-established public health informatics framework for delivering an integrated information system infrastructure that enhances the effectiveness of public health emergency preparedness [26].

## 3. The G3DMS Architecture

**Figure 1:**
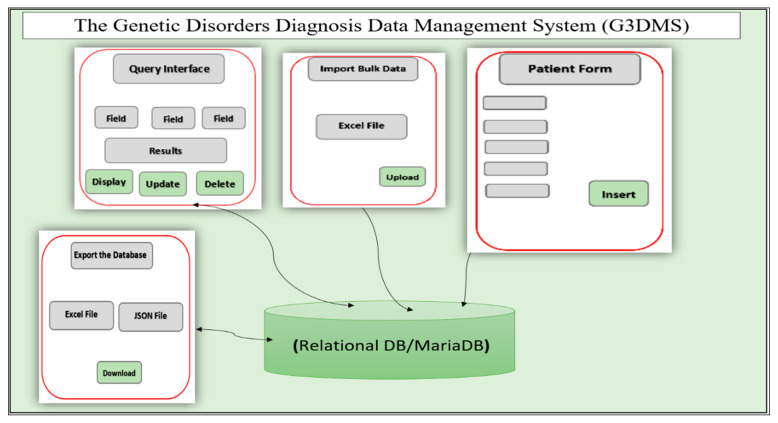
The Genetic Disorders Diagnosis Data Management System (G3DMS) architecture.

The purpose of the G3DMS is to manage patient data collected during the diagnosis process to be used for decision making and research studies. Therefore, we define the structural components of the G3DMS to serve the objectives of data collection, data storage, and reporting, where data is collected and processed for both an individual patients level or a group of patients, taking into account a standard format for entering new data and transferring data from old systems. Figure 1 displays the components of the G3DMS: (A) a relational database MariaDB as a backend for data storage; (B) the application interface which includes a web-based interface with authorized access to the following features:

- Electronic data capture to standardize data entry of patient information;
- Import bulk data in Excel format and map the content of the file to the database using a customized mapping method;
- Query interface to display, update, and delete patients’ records, as well as build customized queries
- Export the content of the database to an Excel file and JSON file format.

## 4. The Design Methodology

The system architecture presented in Figure 1 is implemented using a two-fold method: first, a case study from the Saudi context is used to understand and analyze the current system limitations and propose a new system design model; second, examining the design and implementation of the proposed system using the Barker method for the full system development process.

### 4.1. Case Study

#### 4.1.1. Aim of the Case Study

The objectives of this case study are two-fold: (1) analyze the health information system (HIS) of a genetic research center in Saudi Arabia, the Princess Al-Jawhara Center of Excellence in Research of Hereditary Disorders (PACER-HD) at King Abdulaziz University (KAU) hospital, Jeddah, and (2) use the findings to propose a system design that could enable both clinicians and healthcare researchers to collect, manage, store, and use genetic data for decision making and research purposes.

#### 4.1.2. Description

The PACER-HD provides several free medical services related to genetic disorders, including The Genetic Disorders Clinic which receives referral cases from different departments of King Abdulaziz hospital and from neighboring hospitals in the region as well as neighboring areas, where genetic cases are diagnosed at all ages; The Down Syndrome Clinic which is the only clinic in the Kingdom that provides the following services in only one visit: a full examination, genetic counselling, and follow-up, as well as linking them to rehabilitation centers; The Genetic Counselling Clinic which provides detailed genetic counselling services to families, such as consanguineous marriages, families planning on going for preimplantation genetic diagnosis (PGD), and families requiring genetic analysis of samples. In addition to medical services, the PACER-HD also established a laboratory to generate stem cells to conduct research projects and answer questions in the field of biological studies. Furthermore, they also have a molecular biology laboratory which is run by research teams for various projects and adheres to international standards and uses the latest techniques such as sequencing and DNA/RNA extraction to obtain accurate results and a cytogenetics laboratory where various analyses and experiments such as chromosome analysis, microarray, cell culture and the FISH technique are performed [27].

#### 4.1.3. Methods of Gathering Requirements

We brainstormed with the director of the center and the clinicians participating in the research who were able to define the process of diagnosing genetic disorders to gather the requirements of the processes performed in PACER-HD and determine the data required to support the process. This helped us to perceive the problem from the perspective of healthcare professionals who communicated their problem and their needs directly to us without any ambiguity.

#### 4.1.4. Problems with the Current System

Based on the brainstorming discussion with the Director of PACER-HD and the professional staff, a summary of the response to our interview is as follows. First, the present system lacks the basic capacity to handle a simple research question on genetics. Second, although the available system allows researchers to make a single selection for a specific condition, it does not provide even a simple query answer involving more than one field (vertically) as the patients’ data are stored in a flat-file structure with no relation between the fields. Third, the data gathered from paper-based documents and patient information are manually entered into the HIS by researchers using traditional Excel spreadsheets.

#### 4.1.5. Current Process Model

**Figure 2:**
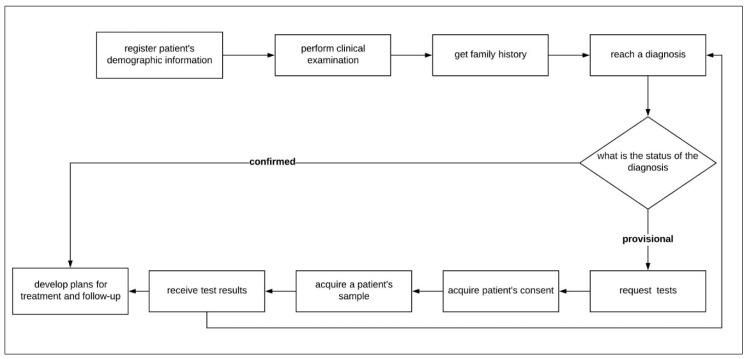
The basic process model for a new patient’s diagnosis.

The basic process model at the PACER-HD is presented in Figure 2, in which the first step is to collect the demographic information, the clinical phenotypes, and family history. The next step is to make a diagnosis to determine if the case is identified. If the diagnosis is confirmed on the spot, the patient undergoes a treatment plan directly. Otherwise, a diagnostic test is requested to confirm the decision. The patient must give their consent before the sample is taken for the diagnostic test. The results are delivered to the clinic, and if the diagnosis is confirmed, a treatment plan for the patient is prepared. All this data must be recorded for every patient in the system of the PACER-HD with any additional documents or literature useful for diagnosis or research.

#### 4.1.6. The Data Elements

The decision to include various data elements was made in discussion with the experts in this field, the PACER-HD director and the professional staff, to determine the essential fields required for the system and the research studies in this area. They summarized the required data elements that they wished to include in the prospective system. Table 1 displays the data required by the center, such as patients’ clinical and demographic data, non-genetic investigation results, and the genetic testing results. Note, the observation data is not applicable in this case as the local laboratory in the genetic clinic only collects patient samples for genetic testing to be shipped abroad to a diagnostic laboratory, and later receives the results and hand-delivers these to the patient’s physician. The genetic test types that can be used for the diagnosis are provided by the laboratory staff at the PACER-HD, as shown in Table 2. Usually, patients’ data can be provided in the form of Excel files and the test results can be submitted in the portable document format (PDF) or image format (JPG).

### 4.2. The Barker Design Methodology

**Figure 3:**
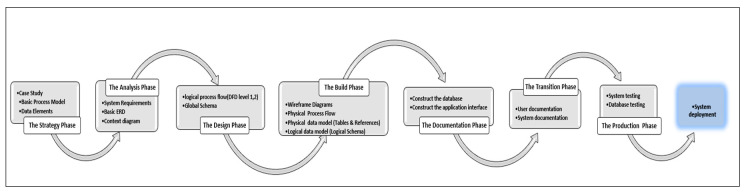
The design lifecycle of the G3DMS using the Barker method.

We adopt the Barker design methodology in the G3DMS for the full cycle of the system development process and the design of the database. Both the process models for the application interface design and the data models for the database design will be considered during the design lifecycle. The Barker method extends the traditional method (requirement analysis phase, data modelling phase, and normalization phase) with more steps to better organize the design effort. The Barker method involves seven stages, as illustrated in Figure 3: Strategy, Analysis, Design, Build, Documentation, Transition, and Production [15]. At the strategy phase, the focus will be on the data and application requirements depending on the process model and the data elements of our case study, the PACER-HD center where the proposed system will be implemented. During the analysis phase, the requirements for the clinical genetic research database will be determined by investigating the research requirements in terms of data, queries, and processes. The analysis results will be displayed using the entity-relationship diagram (ERD) and will show the detailed entity attribute relationships in the global schema, as well as the data flow diagrams (DFDs) in multiple levels for system processes. In the design phase, the logical schema, which was converted to tables and references, as well as the physical process flows which were converted to wireframe diagrams, will be prepared based on the outcomes from the strategy and the analysis phases. In the build phase, the logical schema developed in the design phase is used to construct tables and references, and the application interface prototype will be coded into actual web pages. Documentation of all stages of database design and application interface can be extracted from the diagrams and structural drawings in addition to the preparation of a user guide for system use. The transition phase will encompass the validation testing of both the database and the complete system in the development environment before deployment. Finally, the production phase will include the implementation of the system online to be accessed by the PACER-HD end-users after moving the organization legacy data.

#### 4.2.1. The Strategy Phase

The design and development of a data management system for an organization requires consideration of all the information involved in the business processes. Therefore, the requirements definition is an integral step in the data modelling process to specify the data content of the database [17]. Hence, we rely on a customized strategy to design a data management system for the diagnosis of genetic disorders employing the major process that results in generating patient’s data that can be useful for research if captured and stored appropriately. Therefore, our strategy is to cover the following points using appropriate sources from the PACER-HD case study:

- Highlight the current services provided by the genetic clinic or research center.
- Identify the processes performed in the center and understand the tasks and the role of the researcher and the effectiveness of the current system in meeting the research process requirements.
- Identify the data elements required to perform the processes.
- Determine the research process requirements and the researchers’ expectations of the prospective system in terms of dealing with data collection, management, and analysis.

##### Context Diagram (DFD Level Zero) 

**Figure 4:**
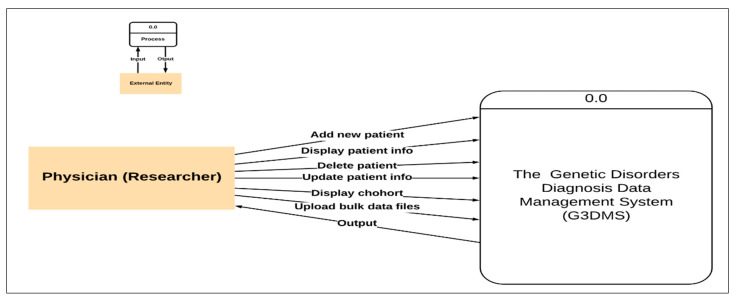
A context diagram (data flow diagram (DFD) level zero).

The basic process model for the primary process performed at the PACER-HD is illustrated in Figure 2. From this, we can determine the main entity is the patient and the primary user of the system who performs all data entry into the system is the physician. Therefore, the next step in the development of the process models is to draw the data flow of the diagnosis process identified in the case study. Figure 4 shows the context diagram or level zero data flow diagram, which graphically represents the whole system as a single process, emphasizing the interaction of the external entity or the physician with the system. All requests and orders from the physician to the system are presented as an input operation and the system response as an output operation. The DFD level zero diagram is foundational to the development of the logical flow of data through a system to perform any task required from the system, for example, add new patient, display patient information, and delete patient records.

##### The Basic ERD

**Figure 5:**
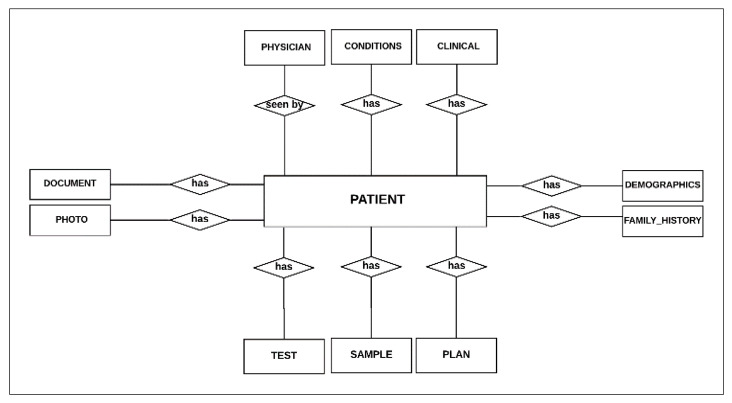
The basic entity-relationship diagram (ERD).

The data elements (entities) are derived from the diagnosis process elements and data required by the PACER-HD in Table 1. Entities or main objects that play an essential role in the diagnosis process and their primary relationships are described in the basic entity-relationship diagram. Figure 5 shows the basic ERD, which only displays the connection between objects without emphasizing the degree of the relationship. The patient is the central entity in the design, as all other entities must have a direct link with the patient. For example, the patient must have demographic information and family history; however, the patient may have several conditions, clinical phenotypes, tests, samples, and treatment plans.

##### The System Requirements

The focus of our strategy is to use the data from the case study and extract a plan for preparing a process model and data model in addition to the system requirements which will be discussed according to the current issues with the PACER-HD system. Determining the expected system requirements in terms of the rules governing data entry and storage in the database will help define the requirements of the database and the application interface. The aim of the G3DMS is to help discover the genetic diseases prevalent in the region with the related risk factors, especially those that can be prevented. Therefore, in addition to assisting the registration of all patient data in the diagnosis process, one of the basic requirements of the system is dealing with genetic research studies and answering biological and statistical questions using relationships between conditions and family history to answer questions such as the association between maternal age with genetic disorders in local population; and allocating cases with both genetic disorders and non-genetic diseases. The current process of answering these kinds of questions is through exhaustive manual research. Researchers and medical practitioners expect the database to be able to accommodate both clinical and genetic data, including unstructured data such as photos, patient consent forms, patients’ pedigree chart, literature and patient reports. Therefore, the database should be able to provide reports and answer queries such as:

- The common genetic diseases among patients in the database.
- The presence of consanguinity patients with specific genetic disorders.
- The relation between mothers of advanced maternal age and specific chromosomal disorders.
- Group patients with common phenotypes regardless of their diagnosis.
- Search and compare a newly discovered mutation with the mutations in other patients.
- Group all cases that underwent specific diagnostic tests and compare their results.

#### 4.2.2. The Analysis Phase

At the strategy phase, all the requirements are gathered from the case study, the basic process model, and the data elements. The results are identifying the data flow between the processes and entities presented in the context diagram (DFD level zero), the relationships between the entities as shown in the basic ERD, and the system requirements. In this phase, both process requirements analysis and data requirements analysis are performed to identify the best data modelling design option for the database based on the analysis outcomes. The results of this stage are the logical process model for the interface design and the conceptual data model or the global schema for the database design.

##### Process Requirements Analysis

Given the comprehensive description of the activities of individuals within the center for the diagnosis process, the data flow is the foundation for the application interface design. First, we define the system properties that we consider necessary in building this system according to the requirements of the operations undertaken in the center.

System specifications:

- The system must provide authorized access to users (doctors/researchers), giving administrators greater privileges to manage access control.
- The system should allow the capture of patient clinical data as well as genetic results and reports.
- The system must provide a seamless, web-based user interface for end-user interaction.
- The system must allow the patient’s record information to be viewed, searched, and modified.
- The system should answer the anticipated queries and enable the researcher to build and customize their questions using the existing fields from the database, avoiding the complexity of query writing.
- The system should allow old data that may exist in the form of Excel files to be imported and integrated.
- The system should follow standardized data collection and storage procedures to enable collaboration and data sharing with other systems.
- The system should allow the export of data from the database in a useful format for integration and dataset sharing.

Based on the requirements, the G3DMS needs to accommodate patients’ clinical and genetic data for care and research purposes suitable for any hereditary disease’s clinic or research center. The relational database model will suit the design of the database for this system according to the requirement for storage and data management for research. Features such as integrity constraints and normalization will further improve the relational model by simplifying data management and data retrieval and making it possible to answer both simple and complex queries. In addition, it supports multiple users and is easy to modify without affecting the entire model body.

**Figure 6:**
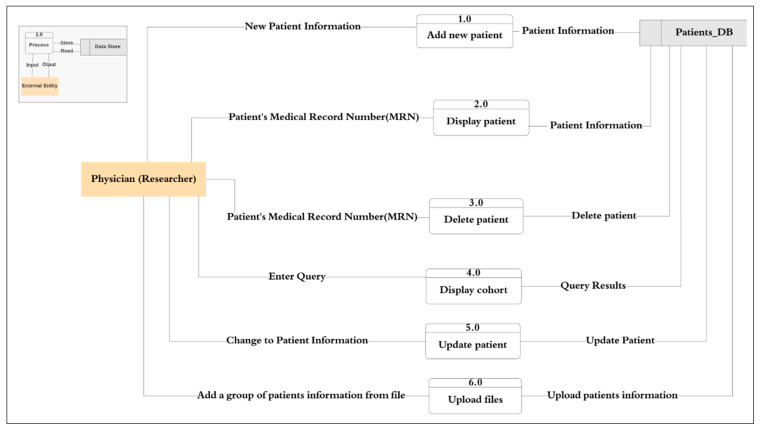
The logical process flows for DFD level 1.

Logical process flow:

Based on the system specifications and the context diagram (DFD level zero), we prepared the logical process flows for the sub-processes and delivered the data flow diagram (DFD level 1) presented in Figure 6, which outlines the primary processes performed by the physician (researcher) such as process 1.0 to add a new patient, and process 2.0 to display a patient. Figure 6 shows the data flow between the external entity, the researcher, and each process is represented as an input and output procedure, and between the process and the patient database as a store and read operation. However, for further analysis, it is necessary to decompose the processes. Therefore, the next step is DFD level 2, which goes one step deeper and shows a more detailed sub-process. For example, the process of adding a new patient to the system is subdivided into sub-processes such as 1.0 to register demographic information, 1.1 to register clinical phenotypes, and 1.2 to register family history showing the data flow between the physician and the system. In every sub-process, the data flow towards the database is presented to show the data storage to keep the data which is necessary for the performance of other processes.

##### Data Requirements Analysis

Based on the data elements provided in Table 1 from the case study of PACER-HD and the primary ERD in the strategy phase, we define the data elements required for PACER-HD database modeling. There is a need to group fields with shared characteristics into categories to form new entities to minimize the number of tables. For example, DEMOGRAPHICS arranges all the patient’s information such as Medical Record Number (MRN), GN, Name, DOB, Gender, Nationality, Contact and FAMILY_HISTORY hosts all the family history associated with genetic information such as Inheritance Pattern, Consanguinity, Mother’s Age, and DNA. There is also a need to break down some fields into more entities to increase the ability to answer more precise quires. For example, GENETIC INVESTIGATION and NON-GENETIC INVESTIGATION do not include any information about the test type, the description, the sample type, or the result. Moreover, new fields/entities are added to include new areas that are required to answer general research questions such as the CONDITION name and CLINICAL phenotype description in the diagnosis process. This will help with the research analysis and answer queries related to this area.

Identify entities and attributes:

We break down the research processes carried out in the PACER-HD clinics into small elements which are mainly about the patient and diagnosis and may include other supporting elements such as genetic and non-genetic tests to achieve the relevant diagnosis. The data elements identified in Table 1 can be organized into entities and attributes with the relationships between them forming a conceptual representation. We follow three guidelines for classifying entities and attributes [28].

- Entities should contain descriptive information.
- Multivalued attributes (an attribute that can have more than one value associated with the key of the entity) should be classified as entities.
- Attributes should be attached to the entities they describe.

Based on the guidelines for classification, the data elements, and considering the patient-centered design, the PATIENT entity is the central entity, and all other entities have a direct relationship, such as DEMOGRAPHICS, FAMILY_HISTORY, DOCUMENT, PHOTO, CLINICAL, CONDITIONS, PHYSICIAN, TEST, SAMPLE, PLAN:

- (One-to-One) relationship between PATIENT and DEMOGRAPHICS, FAMILY_HISTORY entities (each patient has corresponding demographic and family history details, and each demographic and family history belong to a single patient).
- (One-to-Many) relationship between PATIENT and DOCUMENT, PHOTO entities (each patient may have many documents and photos, and in return, every document/photo should belong to a single patient).
- (Many-to-Many) relationship between PATIENT and Entities such as (CLINICAL, PHYSICIAN, CONDITIONS, SAMPLE, PLAN, and TEST) for example (each patient may be diagnosed with many conditions and any condition may be detected in many patients).

Table 3 shows the entities and the data elements that describe the entity characteristics called attributes, in addition to the identifiers, which are the primary keys used to uniquely identify each entity. For instance, we used patient MRN as the primary key for the PATIENT.

**Figure 7:**
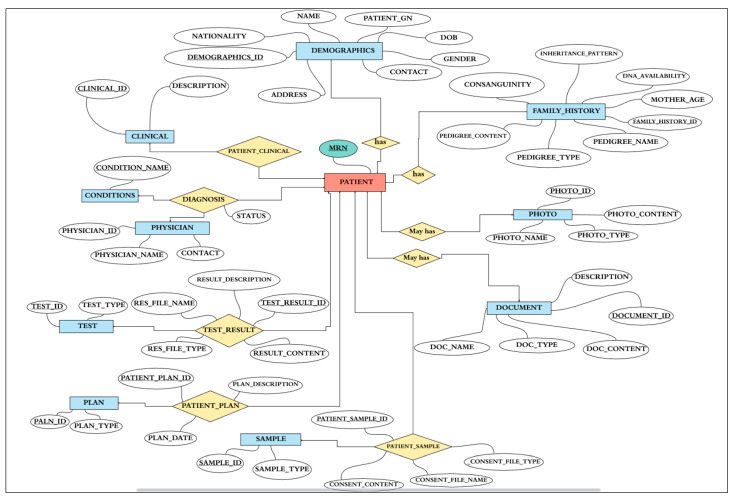
The entity-relationship diagram (ERD) (The Global Schema).

The Global schema:

The entity-relationship diagram (ERD) is used to satisfy the data modelling objectives and develop the conceptual model or the global schema which will be used to construct the physical database structure. As shown in Figure 7, the ERD outlines all the details of entities with their attributes defined in the previous section as well as identifiers and constraints. In the diagram, the primary keys are underlined to distinguish them from the attributes of the other descriptors. The cardinality was specified by minimum and maximum values (one, many, one or many, and zero or many). This is to show the constraints, or a restriction placed on the data to ensure data integrity. For example, for a patient to be included in this database, there should be at least one condition (one or many); however, a patient may or may not have documents (zero or many). The ERD also shows two types of relationships:

- Binary relationships between the PATIENT entity and another entity such as DEMOGRAPHICS, FAMILY_HISTORY, DOCUMENT, PHOTO, SAMPLE, TEST, PLAN, or CLINICAL;
- Turnery relationship between the PATIENT, PHYSICIAN, and CONDITIONS entities.

#### 4.2.3. The Design Phase

The outputs of the analysis stage is the logical process flow which includes the DFD level one and two as well as the conceptual data model or the global schema. In this phase, we develop the logical data model or logical schema from the conceptual model and then convert the logical schema to the physical data model, i.e., construct the database tables and references. Furthermore, we proceed towards the design of the application interface depending on the logical process models and prepare the physical process flow and the interface wireframe diagrams.

##### The Logical Data Model

**Figure 8:**
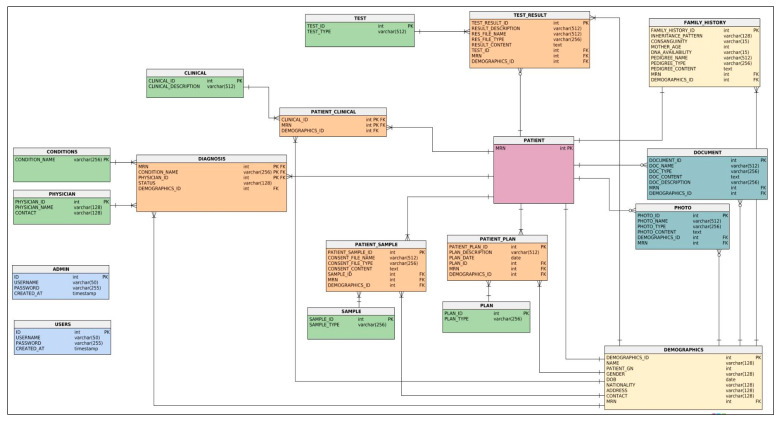
The logical schema.

Throughout this phase, the focus is on developing the physical model by converting the logical schema that has been built based on the entity-relationship (ER) diagram for data requirements specification and the conceptual model defined in the previous two-phase strategy and analysis. The data model described in the ERD in Figure 7 is converted to suit the DBMS, so entities, attributes, and relationships must be converted into tables and fields. Therefore, any entity will be converted to a table, its attributes will become fields, and its primary key will become the primary key of the table. In the case of the many-to-many relationship between two entities (binary) such as PATIENT and SAMPLE, a third table will be created with the name PATIENT_SAMPLE. Similarly, in the case of many-to-many between three entities (turnery), for example PATIENT, PHYSICIAN, AND CONDITIONS, a third table will be created with the name DIAGNOSIS by combining the three primary keys from the participating entities. The normalization technique is applied to refine the design to remove duplication (uncontrolled data redundancy) that may occur in tables to eliminate the problem of modification anomalies (while adding, deleting, and modifying records in a table). The logical structure of the database (schema), namely the structural representation of what is in the database is presented in Figure 8, which clearly defines the tables (entities), the fields (attributes) in the tables, the primary and foreign keys, and the relationships between tables.

##### The Physical Data Model

**Figure 9:**
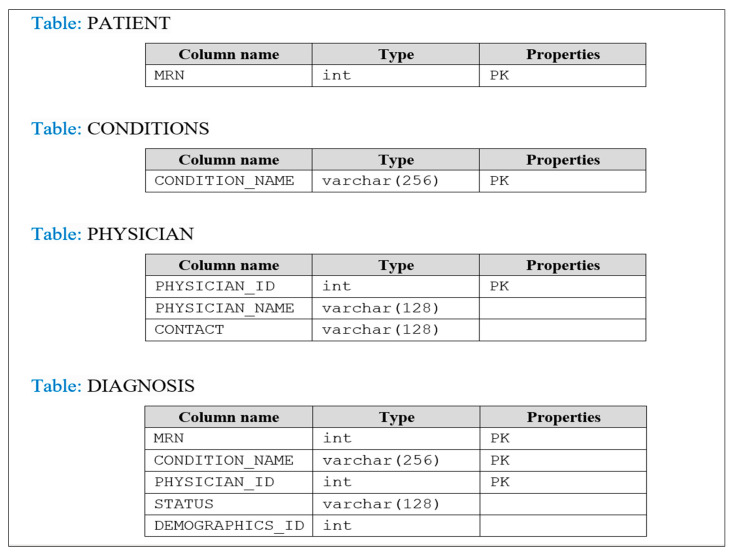
Converting the logical schema to physical model/tables.

The logical schema in Figure 8 is converted to a physical model where entities are mapped into tables, instance into rows, and attributes into columns. Each table contains the column names and its specified data type, and the primary keys are also determined for each table which can be a single or composite key. References are used to describe the relationships between tables recognized by foreign keys; references facilitate the indexing mechanism for efficient access to the data. Figure 9 shows the physical representation of a many-to-many relationship as tables, for example the DIAGNOSIS table with its composite primary key (MRN, CONDITION_NAME, and PHYSICIAN_ID) and their respective tables. However, the linkage between the entities of a one-to-many relationship can be represented using a referencing key or foreign key.

##### The Physical Process Flow

In addition to the logical database schema, the product of this stage is the physical process flow which is based on the logical process flow in the analysis phase. The focus of this model is on the detailed description of the system and user interactions. The physical process flow determines how the user interface will be designed and how forms are constructed to collect data from the user and submit it to the database. For example, the process of adding a new patient is logically illustrated using the data flow diagrams. The physical process flow shows a comprehensive view from the perspective of the application interface interaction, including all responses in the form of messages to each user action.

##### Interface Design

**Figure 10:**
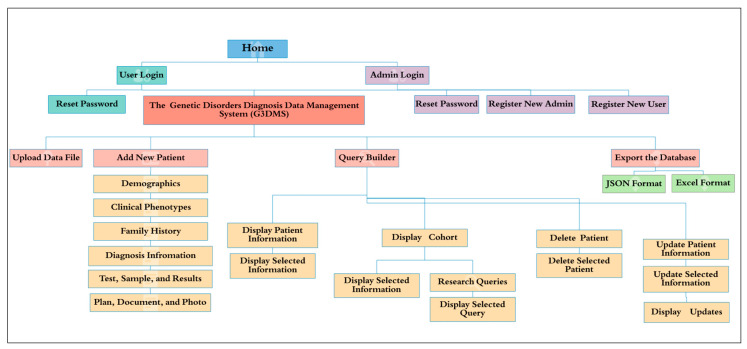
The site map of the G3DMS.

The processes flow models are translated into the actual structure of the application interface, which will be developed accordingly. The site map in Figure 10 illustrates the plan for the organization of the web pages according to the intended user action to perform a specific task within any process flow. This hierarchical representation clearly shows the parent–sibling relationship between the pages of the website. The home page provides an authorized entry to the system according to the role of user or administrator. The admin web page provides access to pages to reset passwords and register new users or admin, as access to this system should be approved by the appropriate organizational authority. The user also can maintain his account and has access to the reset password page. Users who have both roles can access the G3DMS main page, which can provide access to four pages: Add New Patient, Query Builder, Upload Data File, and Export the Database. This page allows the user to move sequentially to the next page to fill in all the patient data using text boxes, starting with the demographic information page to the plan, documents, and photos page. The query builder page provides access to four pages, these being to display the selected patient’s information, display a cohort with common characteristics and research queries, delete a patient, and update patient information.

**Figure 11:**
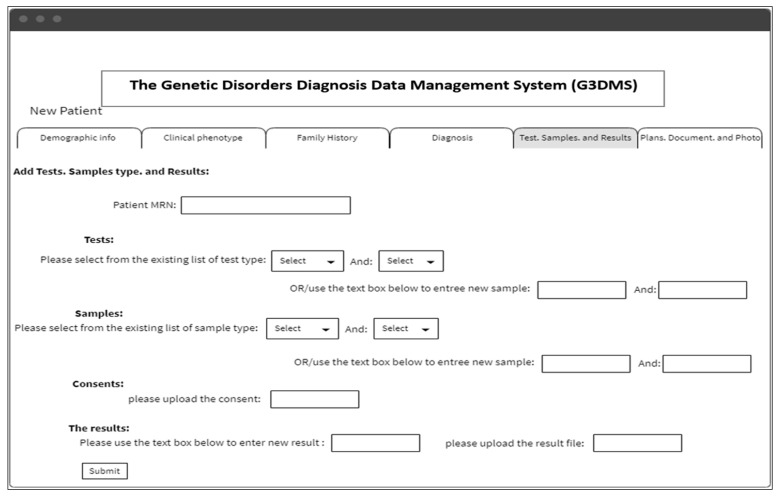
Wireframe diagram for adding new patient: tests, sample, and results page.

Wireframe diagrams:

While the site map shows the organizational and hierarchical presentation of the web pages, wireframe diagrams outline the layout and element functionality within the page and the site navigation details. Figure 11 presents a sample page of the interface using the wireframe diagram. An example of adding new patient data is provided in the layout; the page for filling in the diagnostic tests requested by the physician, the patient sample data with the consent document, and the tests requested with its results can be in the form of text or uploaded as a file.

#### 4.2.4. The Build Phase

Building the database involves the actual creation of the whole system, the application interface, and the database that has been physically designed in the design phase. In this phase, the programming code and the database code are written. The build phase takes place in our chosen test environment to build the initial schema before the implementation in the production environment. First, the choice to build the database using the relational model as specified in the analysis and the design phase according to the requirement analysis and the purpose of the G3DMS will facilitate the data structure and management using the right relational database management system (RDBMS) for such a system. Therefore, the creation of the database will depend on the selection of the storage container or server and the DBMS to run the database scripts. Second, the application interface development option also will affect the entire system performance; therefore, selecting the environment that supports the structured query language (SQL) language as well as the interface programming is very crucial. Thus, in this phase the aim is to choose the development environment that accommodates the design for the back-end database and the front-end application interface, including the user interface (UI).

##### Build the Database

**Figure 12:**
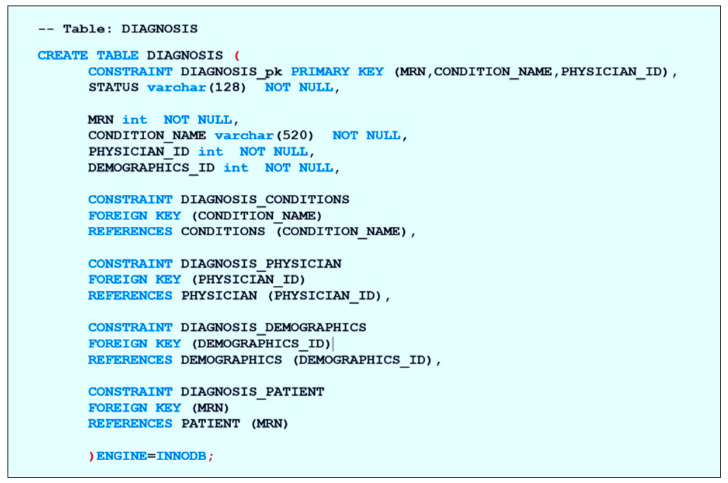
SQL script for creating the “DIAGNOSIS” table.

For the database construction, we implemented the physical data model (tables and references) in the phpMyAdmin server. First, we created the database and then we created the “PATIENT” and the “DEMOGRAPHICS” table, on which all the other tables in the database depend. After this, the independent tables are created, such as “CLINICAL,” “CONDITIONS,” “PLAN,” “SAMPLE,” “TEST,” and “PHYSICIAN,” which store a list of shared information that may be linked to any patient in the database. Then, the patient tables that depend on the previous tables are created, such as “DIAGNOSIS” which depends on “PATIENT,” “CONDITIONS,” and “PHYSICIAN,” in addition to “DEMOGRAPHICS” for easy access to the patient’s personal information. Figure 12 shows the script for constructing the table “DIAGNOSIS” showing the primary key and all its attributes as well as the foreign keys that link the table to its associates. Tables such as “FAMILY_HISTORY,” “DOCUMENT,” and “PHOTO” which are directly dependent on the “PATIENT” table are also created similarly.

**Figure 13:**
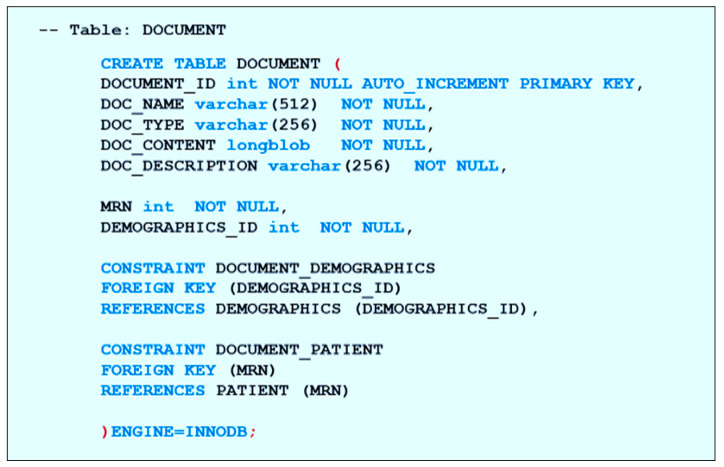
SQL script for using “LONGBLOB” data type in the “DOCUMENT” table.

Data types are stored as specified in the database library in the physical data model, except for the document contents, and the photos are stored as a BLOB data type which is a binary large object. Storing patient data files such as documents, reports, and photos in a long binary large object (LONGBLOB) format allows us to keep the content in the database and retrieve it without any damage. Figure 13 shows the script used to define a document using a name, type, and content in the “DOCUMENT” table.

##### Build the Application Interface

Once the database has been created, the G3DMS application interface is ready to be constructed, considering the user interface layout based on the wireframe diagrams and linkage methods to communicate between the user interface page requests and the database response. Each wireframe design for a specific page is converted to the actual web page using HTML code to create forms to collect data using menus for selection as well as text boxes to insert data. The PHP MySQLi functions allow for connecting and communicating to the MariaDB server using MySQLi driver methods for adding, reading, updating, modifying, and deleting the content of the database. An open-source cross-platform web server solution stack package (XAMPP) platform is also used as a development environment for the front-end using the Apache server as a local host to run the source code for the webpages during the development process [29].

**Web interface pages:**

Login pages:

Access to the system is restricted to authorized users only. Therefore, the first page in the interface is designed to display the login options according to the user’s role and their level of authorization. The system allows logging on as a regular user or as an administrator. Administrators have additional privileges, such as being able to register new users and define their roles. Both users of the system can reset their passwords through the reset password page after logging in.

The G3DMS home page:

The G3DMS home page is an HTML coded page that provides links to the Register New Patient page, the Upload Bulk Data File page, the Query Builder page, and the Export the Database page. It also contains a link to download the user guide in a PDF file for more instructions and the user interaction documentation.

Register New Patient page:

The process of registering a new patient is completed in six steps to facilitate the data entry process and allow the user to focus on each step individually and prevent information clutter. The page structure starts with demographic information followed by clinical phenotypes, family history, diagnosis information, tests, sample type, test results, plans, documents, and photos. The system will allow the user to complete the patient’s data sequentially; each step of the registration process is developed in an individual PHP page with HTML codes to display the forms. First, before entering the data, the system allows the patient MRN to be checked in the database to prevent data duplication and override. The MRN is checked in the PATIENT table, and then a response message is shown if it exists, but if it does not exist then a new record is created in the PATIENT table, and by submitting the form the data is sent to the other designated patient’s table.

Upload Bulk Data File page:

The G3DMS offers another method of data entry, especially for migrating old data or data collected offline for research. The bulk data upload page contains two options: download the Excel template to fill in the patients’ data offline, and the upload button to import bulk data using the Excel template.

The Query Builder page:

This page is the main access page for all kinds of simple, complex, and advanced queries. It provides links to the following pages: Display Patient Information page, Display Cohort pages, Update Patient Information page, and Delete Patient page. Each option links to the page that performs the selected task. The Display Cohort page provides access to perform more advanced queries through the Display Research Queries page.

Export the Database page:

This page allows users to obtain datasets from the database in the form of Excel files and JSON files. The page provides two options: Export to Excel and Export to JSON buttons. By clicking on the button, the data will be downloaded to the user’s computer in the requested format.

Building features:

The G3DMS aims to deliver an easy-to-use user interface equipped with features to facilitate both patient care and to produce quality datasets for research. Therefore, several aspects are considered while building the application interface to ensure ease of use, data quality, research results accuracy, and an interactive nature.

**Figure 14:**
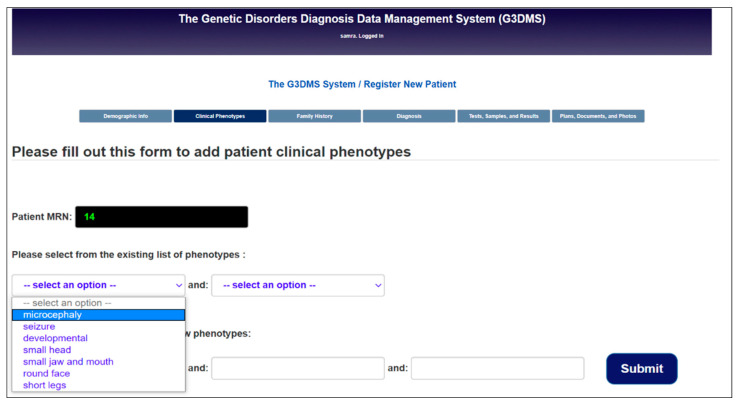
Progressive lists example: clinical phenotype menus.

Progressive lists:

As the users enter new data for the first time using textboxes, the self-developing dynamic lists are automatically updated and available to be used for data entry. The main advantage of this feature is to reduce data duplication which affects data quality, hence playing an important role in the accuracy of the research results. It is less time consuming for users to enter data, so they can focus more on the care process instead of data entry. The progressive lists combine common characteristics such as phenotypes, conditions, physicians, sample types, tests, and plan types which can be shared by multiple patients. Figure 14 shows an example of a progressive list for patients’ phenotypes which will be displayed when registering a new patient. This menu will be filled in by inserting new phenotypes using text boxes which constitutes an alternative method of data entry. Then the submitted data will be checked by PHP MySQLi Functions, and if the inserted value does not exist, it is inserted in the CLINICAL table and referenced in the PATIENT_CLINICAL table using the ID. However, if the value exists in the table, then the CLINICAL_ID is retrieved and linked to the PATIENT_CLINICAL table. For multiple phenotypes, the entry is stored in an array and is then retrieved using foreach to access all the values. To display the list, as shown in Figure 14, we use a PHP code to read the table using the SELECT statement and then display the results inside the HTML <select> as an <option>.

Interactive menus:

The page structure of the G3DMS development should be clear and avoid ambiguity. Therefore, we focus on reducing the number of unnecessary elements not related to the task at hand. To do this, we used the jQuery function to manipulate the appearance of menus on pages specifically designed to answer research questions. First, we added the jQuery function as <script> with the HTML code. Next, we defined each select list as a variable inside the function, and then used the hide () function to hide all the lists except those required by the user.

**Figure 15:**
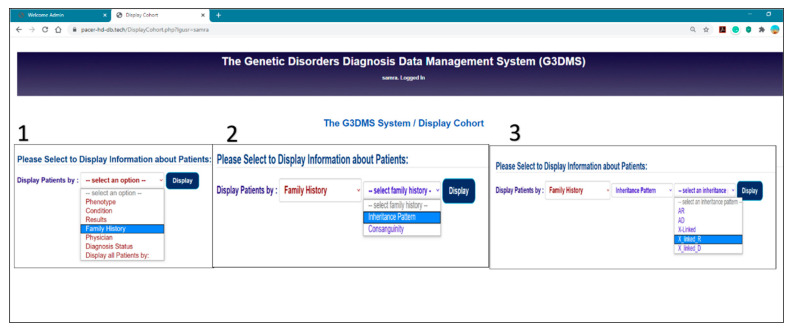
Interactive menus example in the display cohort page: three-option menu display.

This feature is implemented in the Display Cohort page which displays a group of patients with common characteristics; first, the user selects from the first dropdown list to choose the main category (phenotype, condition, results). Then, according to the user’s selection in the first list, the system displays a dynamic list for that option. Figure 15 depicts an example of a user selecting Family History from the first menu, at which point another menu is displayed for the options (inheritance patterns or consanguinity) and depending on the user’s selection, a third menu appears which displays a dynamic list of content from the database.

Customized alert messages:

The system provides an interactive environment for the user as any action a user performs is considered in a case of success or failure. Accordingly, customized messages are designed to extract the message from the user’s query. The response will be relevant to the user’s action and provides a satisfying answer. These types of messages appear in multiple cases in the search queries section, Query Builder pages. We implement a JavaScript function within the PHP to manage the alert message.

Import functionality:

The G3DMS offers another method of data entry in addition to the patient’s registration forms, which is to upload bulk data aimed at migrating old data or data collected offline for research. The Excel file template is used to upload bulk data all at once. The template sheet provides the fields required by the database, which need to be filled with patient data in each row.

**Figure 16:**
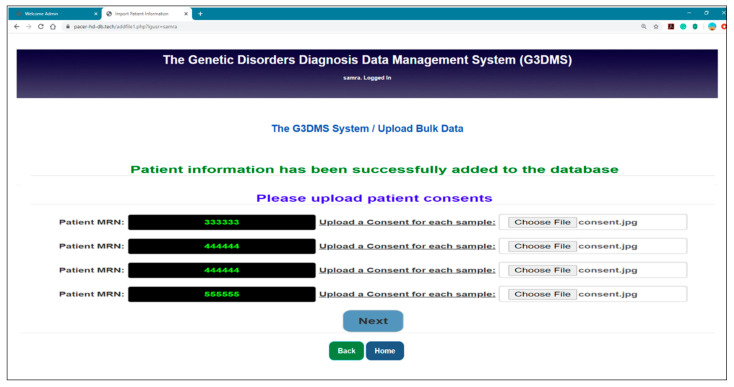
Upload bulk data: patients’ consents for YES value fields.

Mapping method:

The upload button allows the loading of the Excel template and triggers the mapping process using PHPExcel classes as follows:

require_once ‘Classes/PHPExcel/IOFactory.php’. The getActiveSheet() function is used to read the active worksheet and getHighestRow() and getHighestColumn() to get the content from each row and column into an array of objects and then allocate each value to its specific table location in the database. First, all the contents of the MRN column are stored into an array, then the array is looped and each MRN is checked to determine if it exists in the database. If it does not, then the patient record is inserted in each field in its associative table, or else this patient is skipped. Within a patient “row” record, it is necessary to verify the presence of common field values such as conditions, clinical phenotypes, and tests, in their separate table before inclusion, and then they are linked to patient tables. However, in the case of a multivalued column which should be separated by a comma, for example, a patient with two conditions, the mapping method will recognize the separator and insert each condition individually in the condition table after checking for its existence. If it exists, then the condition ID is retrieved and it is referenced in the patient DIAGNOSIS table or else the condition in the CONDITION table is inserted, and the ID is retrieved and linked to the DIAGNOSIS table. We follow a rule-based mapping technique for some columns, which are an image or file type, such as the patient (consent, results, pedigree chart, photo, and documents). YES should fill each field if the patient’s document is available or NO when there is no document for this field. All fields with text, date, or a number value will be inserted automatically, but for fields that require uploaded files and images, the system displays the upload options in each column for all patients on an individual page if the value is YES. Figure 16 shows the page for uploading the patient’s consent filled with the YES value only, but, in the case of NO, the system will discard this field and fill it with NULL.

Export functionalities:

The G3DMS provides a customized mapping method for exporting the content of the database to an Excel file to be used for further research within the user’s local organization. The database content is also downloaded in a JSON file format, which can be integrated with other systems and Saudi open datasets. Only patient data which is useful for further research will be exported. Other data, such as a patient’s treatment plan and the doctor’s information, are not required. As the intention of the JSON file is to contribute to the research community outside the organization, privacy rules will be applied, such as removing a patient’s identifiable information that is linked to the patient’s identity.

1. Mapping method for exporting to Excel

To develop the method to export the Excel file, we use a header function in PHP to create a new Excel file as follows:

header (“Content-Disposition: attachment; filename = The_G3DMS_SYS.xls”);

Then, the content is streamed from each table in the database (using foreach to navigate through the entire table), to be placed under each specified column header. A bar “|” is used as a delimiter in case of multivalued attributes such as clinical phenotypes, samples, and tests. The exported data is restricted to the organization that owns the data due to the provision of a patient’s identifiable information.

2. Mapping method for exporting to JSON

We develop a dedicated method for read-only confirmed diagnosis cases and apply a de-identification method to remove the identifiable primary key, the MRN, to a new hashed key. Therefore, the data extracted can be used as valid datasets in the genetic conditions of the Saudi population without any concern about data privacy. First, a JSON file is created using the PHP header() function:

header(“Content-Disposition: attachment; filename = Patients_Detail.json”);

Then, the extracted dataset is stored in an array of objects to be converted to a JSON representation using the json_encode() function. Next, we used the echo statement to display the resulting encoded data into the created JSON file as follows:

echo json_encode($json_array,JSON_PRETTY_PRINT);

Files and images management:

One of the critical requirements of the G3DMS is the ability to accommodate patient-related documents, such as consents, photos, pedigree charts, results, reports, and research papers. Therefore, our focus is to deliver a secure method for uploading, storing and retrieving this type of data. We prefer to store images and files in the database as a binary object BLOB instead of in a file system storage. As these data files can be used in research queries, they need to be stored in the same database with the related patient’s data to maintain consistency and data integrity. First, after submitting the uploaded file, then PHP $_POST method passes the content to the PHP file to get the file name, type, and content to be stored in three separate variables, for example, storing the pedigree chart image for a patient:

$pedname = $_FILES[‘ped’][‘name’];

$pedtype = $_FILES[‘ped’][‘type’];

$ped_Data= file_get_contents($_FILES[‘ped’][‘tmp_name’]);

$pedContent= addslashes($ped_Data);

Simple query:

The G3DMS provides query services in multiple forms. Simple queries are used during patient registration, checking new entries such as patient MRN, phenotypes, and conditions using a selected statement and checking the results if empty then performing insertion, otherwise returning messages such as in the case when the MRN is not new and creating patient-related tables and referencing the ID of the existing variables. A simple search option is also used to search for a specific patient using the MRN and displays the patient information (demographic information, clinical phenotypes, diagnosis, family history, samples and consents, tests and results, plans, photos, and documents). This can be achieved using a simple select statement, and then the results are displayed using an HTML table as illustrated in the display results section. Similarly, a simple search operation is performed to delete a patient for the given MRN, then the demographic information for this patient is retrieved to double-check their identity and confirm the delete action to remove all the patient’s records from the database. The following is an example of a simple query for checking the MRN to delete, update, or display the patient information.

**Figure 17:**
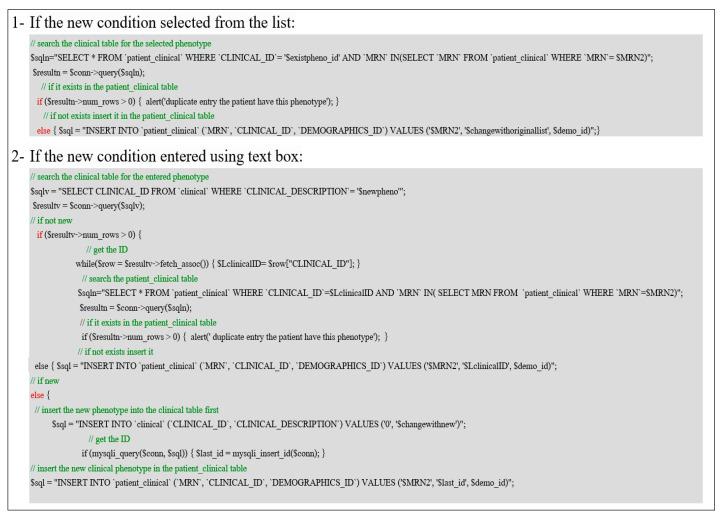
The script for updating patient information by adding a new clinical phenotype.

Complex query:

Complex actions such as modifying patient information by removing existing or adding new information require a more advanced method to perform all the necessary inner operations before completing the task. The action of updating the patient clinical phenotypes by adding or removing them will be performed in several steps. Figure 17 shows the script for updating the patient’s information by adding a new clinical phenotype for a given patient’s MRN, and the user can enter the new phenotype from the database list or use the text box to type in the phenotype.

**Figure 18:**
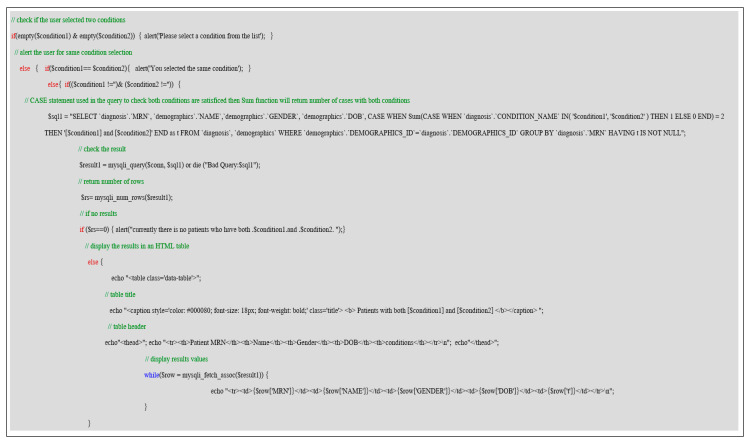
Advanced query to display patients with two selective conditions.

Advanced query:

Specific queries are implemented to answer some research questions displayed in general form and can be controlled by the user selection to be executed in more complex queries. Figure 18 illustrates a common research question where the user can identify two related or suspected conditions in one way or another and show whether a patient in the database suffers from both conditions. We implement two CASE statements, first an inner CASE to check that both conditions are in the patient diagnosis table. If it is satisfied, it returns 1, and we use the Sum function to count the number of elements inside the CASE statement. If the results are equal to two, then both conditions are combined with “and” in another variable “t” and group by patient MRN and having “t” is not null, if all conditions are satisfied the second outer CASE will return the results.

#### 4.2.5. The Documentation Phase

The documentation phase collects the necessary documentation from the system design lifecycle stages and prepares a user guide to support the end-user’s use of the system. This phase is concerned with system documentation and the system’s end-user documentation.

##### System Documentation

System documentation includes documents that describe the actual system structure and all the material involved in the development of the system throughout the design lifecycle. We assign the product of each stage in the system design lifecycle to document part of the system design. Therefore, for requirements documentation, we refer to the strategy and analysis phases. The design architecture documents and the source code can be referenced to the design and build phases. The verification test documents are prepared during the transformation phase.

##### User Documentation

We created a user-centered document guide for the end-users to be delivered through user-friendly instructional content. The aim is to display in detail all the instructions for using the system, how to deal with it, what to expect, how it will react upon errors, and how errors are handled using relevant, informative messages. The documentation is organized according to user tasks and system usage scenarios. We provide screenshots of inputs and outputs with a complete description of each part of the system, including errors and messages to address these errors. User guide documentation (PDF file) is provided as a downloadable attachment via a link in the G3DMS home pages.

#### 4.2.6. The Transition Phase

This phase provides a smooth transition for the physical database from the development and testing environment to production and implementation. The complete system, including the back-end database and the system application interface, is subject to validation testing to ensure the system meets the user requirements. This phrase encompasses data loading, conversion, and end-user training. The end-user documentation is used as training material for the end-user to refer to if they require further instruction to complete their tasks and answer their research enquiries.

##### System Testing

The system supports user interaction with the database through forms with various fields. Therefore, the goal is to test the accuracy of the front-end (database application interface) against the back-end database as well as to verify the accuracy of the database in relation to the way data is stored and accessed in the database. We performed a validation test to ensure that the application works correctly according to the requirements and rules specified in the strategy and analysis phases. Sample data is used in order to test the application interface against the database. The sample data hold real data characteristics except for false MRN and demographic data. Under our supervision, a member of the PACER-HD conducted a system validation test by trying several case scenarios using a testing checklist. However, we performed the database testing using phpMyAdmin as a testing environment. The testing outcomes were very satisfactory.

Interface testing checklist:

- Validate mandatory fields
- Validation error messages
- General confirmation messages
- Validate forms entry to the database
- Importing bulk data upload functionality
- Display patient information
- Display cohort
- Update patient information (Remove, Add)
- Delete patient record
- Research queries functionality
- Export the database

**Figure 19:**
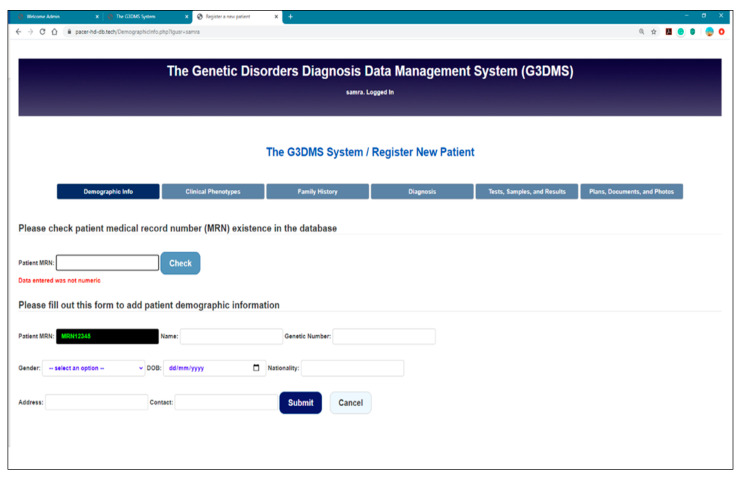
Field entry validation message.

**Figure 20:**
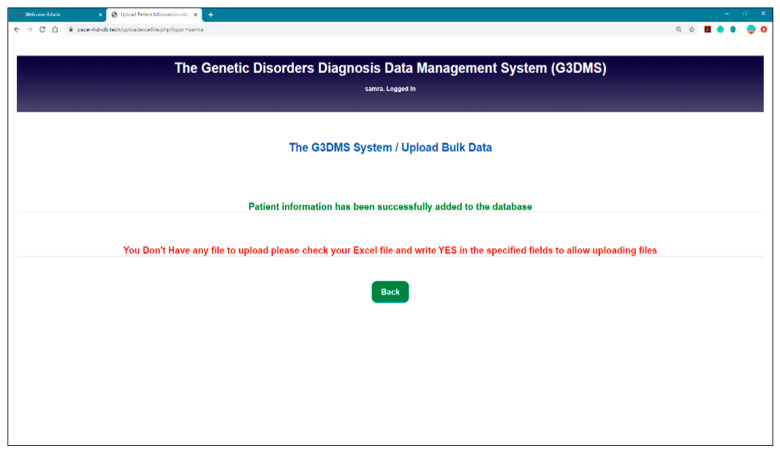
Confirmation messages.

Examples:

Field entry validation messages are displayed for each field to prevent the insertion of blank and non-text variables into the field. We tested the field MRN with a non-numeric entry, and the message “Data entered was not numeric” was displayed as a validation action, as shown in Figure 19. Figure 20 shows an example of a confirmation message posted when the user uploaded bulk data to the database.

##### Database Testing

We used several test scenarios to test the database for accuracy in relation to data storage and retrieval. We used the development and testing environment with phpMyAdmin to run the queries. The following checklist is used to validate data entry, storage, and access in the database.

Database testing checklist:

- Data allocated correctly to the right fields in the database upon successful page submission
- Preserve data integrity when inserting, deleting, updating information in the database
- Data entry in the database is sequential. First, the patient record with the demographic information should be created before filling out any other information.
- Mandated data entry fields saved in primary key positions in the database.
- Check uploaded images and files stored properly in the corresponding fields (name, type, and content) database.
- Check the functionality of the uploaded file of the supported type (PDF) and the image extensions (PNG, BMP, JPEG.)
- Check the validity of the link to display files and images.
- Check the quality of the retrieved image from the database.
- Check query results, specified columns, and correct values are displayed.

**Figure 21:**
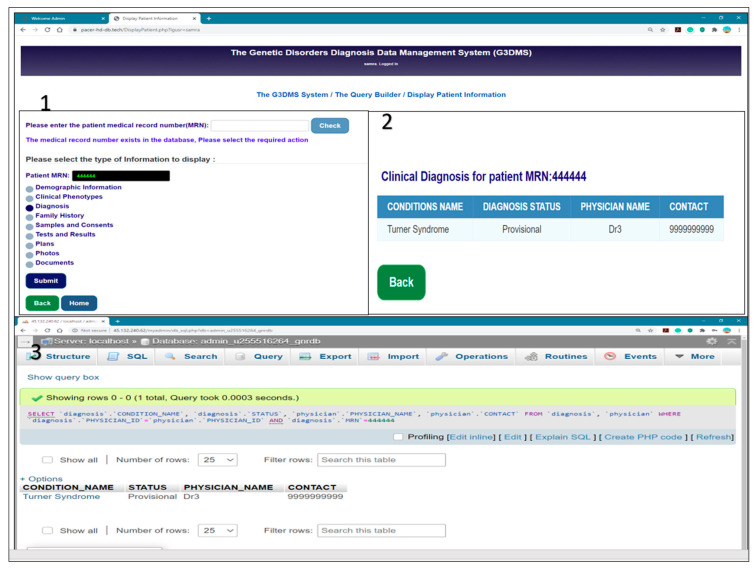
Testing the query (display patient diagnosis information) shows equivalent results.

Examples:

We tested the database for a query using the selection of display patient diagnosis information in our G3DMS interface and wrote the following SQL script for the query in the phpMyAdmin, as shown in Figure 21:

SELECT‘diagnosis’.‘CONDITION_NAME’,‘diagnosis’.‘STATUS’,‘physician’.‘PHYSICIAN_NAME’, ‘physician’.‘CONTACT’ FROM ‘diagnosis’, ‘physician’ WHERE ‘diagnosis’.‘PHYSICIAN_ID’ = ‘physician’.‘PHYSICIAN_ID’ AND ‘diagnosis’.‘MRN’ = 444444;

#### 4.2.7. The Production Phase

The production is the last design stage, which is the application and deployment stage. The content of the system is moved from the test environment, the XAMPP platform, to a live server host using a commercial host for the domain, website and hosting servers. The website https://pacer-hd-db.tech/ is used to access the application interface of the G3DMS online. While the database can be accessed and managed through the phpMyAdmin interface, any changes and updates to the application pages can be done locally at the PACER-HD center and then uploaded using the FileZilla software. The users will have access to the website using their login account as well as access to the database content through phpMyAdmin at their host server.

## 5. Qualitative Evaluation

Good system design is important to ensure system functions work in accordance with their intended purpose. A well-implemented system also affects user satisfaction in relation to sustainable adoption [3]. A poor user and organizational contribution in relation to the design process and implementation results in the potential of HIS not being realized. A lack of clinical engagement is one of the important reasons behind physician resistance, which is one of the most crucial barriers to HIS adoption. Therefore, we adopt two evaluation methods to consolidate our design and ensure the successful implementation of the G3DMS. First, the design and implementation of the system follow the multilevel service design (MSD) approach, which fosters user adoption at the implementation stage [30]. An informatics evaluation framework provides a heuristic for matching the stage of system development according to the system design lifecycle and the level of evaluation [31]. Both methods of evaluation increase user experience with the system, and constant feedback is given in an iterative design lifecycle.

### 5.1. The Multilevel Service Design Approach

Inspired by the successful development and implementation based on a service design approach of a widely used Portuguese EHR, a qualitative study performed after implementation showed that the EHR was considered useful and easy to use, and these results are backed by the widespread usage of the system [4]. Our design and implementation of the G3DMS follow the MSD approach, where all the principles of the approach are delivered through the system design lifecycle. The MSD method depends on the user experience and creating a set of interrelated models that bridge the customer experience and designing the service offering. The MSD process involves four steps: studying the system user experience, designing the service concept, designing the service system, and designing the service encounter [30]. Table 4 shows the steps of the MSD approach with its corresponding action in the G3DMS application. We guaranteed the involvement of the system user throughout the design and implementation process, which will increase user familiarity with and acceptance of the system.

### 5.2. The Informatics Evaluation Framework

Table 5 shows the five stages of the system development lifecycle and equivalent evaluation level according to the Stead et al. framework, as presented by Kaufman et al. [31]. The results of one level of evaluation can be used at a later stage of development or can indicate the need to move to the next level of evaluation. The reason we adopt this model is to allow the evaluation process to reflect knowledge to the development process.

### 5.3. Success Factors

- Physicians are involved in the system planning and design lifecycle (iterative, user provides comments on the trial version, which are used to alter the design model).
- The system users tested G3DMS validity according to their requirements, and it was found to support their workflow.
- Cost-effective: a free system, that is, the department does not have to pay for the system as the author offered free training and assisted with legacy data transformation.
- Subject to future development and support as the center is part of the University hospital, King Abdul Aziz University, where the author teaches.

### 5.4. Recommendation for Successful Implementation (Sustainability)

- Managerial role to assign users to upload the legacy data into the system to save time.
- Assign administrative staff from the department to manage the service provider account payments.
- IT staff to supervise the database and website issues.
- Hold regular meetings with IT, staff, and physicians to discuss issues related to system usage.

### 5.5. The Action Plan Evaluation

The following Table 6 shows the process of evaluating our action plan in terms of (1) Goal, (2) Team Members, (3) Challenges of the current system in PACER-HD, (4) Diagnosis Workflow of the G3DMS, (5) Design and Development Process of the G3DMS.

## 6. Conclusions and Future Work

The major contribution of our research is as follows. This paper highlights current issues faced by the Kingdom of Saudi Arabia in relation to health information systems and health informatics and the resulting impact of such issues in critical areas, such as genetic clinics and research centers. In particular, there is a lack of technical solutions to solve the problem of collecting, storing, and processing clinical data. We proposed solutions to address these issues in the Saudi context based on the current literature. We then implemented these solutions by designing a novel data management system (G3DMS) for the diagnosis of genetic disorders, which supports the health informatics initiative in Saudi Arabia. The G3DMS has three unique support features: (i) the genetic conditions diagnosis workflow; (ii) diagnosis decision-making procedures; and (iii) applicability to research studies. Another strength the G3DMS has is that it is applicable to any genetic clinics and genetic research and can be implemented successfully in any low-resource setting.

Therefore, in this paper, we presented a design and implementation of the G3DMS using the Barker method. In seven comprehensive design steps, we delivered a data management system that applies to any genetic clinic and research center in the Kingdom of Saudi Arabia. A qualitative evaluation of all stages of the design lifecycle was conducted to support the design with the experience of the system user, thus ensuring user satisfaction to obtain a successful and sustainable application. Therefore, the perspective of the G3DMS is to become a standard system for data collection, management and storage to promote data sharing between genetic institutions. In the future, this system is expected to be part of the infrastructure for a multi-system integration platform for the creation of a Saudi national genetic disease database.

## Figures and Tables

**Table 1:** The fields appeared in the Excel file used for data collection of clinical diagnosis process.

Field Name	Description
MRN	Patient’s Medical Record Number which is used to link patients to their hospital information
GN	Genetic Number is given by the Center for tracking purposes
NAME	Patient’s Name
DOB	Patient’s Date of Birth
GENDER	Patient’s Sex
ORIGIN	Patient’s Nationality
DIAGNOSIS	Physician clinical decision of condition according to the patient’s symptoms and signs
IS IT DEFINITE?	Defines the status of the diagnosis if it is provisional or confirmed
INHERITANCE PATTERN	Shows if it is Autosomal Dominant (AD), Autosomal Recessive (AR), X-linked, etc.
CONSANGUINITY	Presents the existence of consanguinity for the patient’s parents (+/−)
MOTHER AGE	The age of the mother at the time of the patient’s birth
DNA-AVAILABLE	States the availability of the DNA test results (Yes/No)
s/b	Seen by or referred to the physician/clinician who is responsible for the patient’s treatments
NON-GENETIC INVESTIGATION	Clinical tests requested to confirm diagnosis
GENETIC INVESTIGATION (RESULT)	Genetic test results to confirm the diagnosis
PLAN	Treatment plan such as follow-ups/rescheduling/referral
PHOTO	The patient’s photos (images)
CONSENT	The signed consent in a PDF format from patient
IMPORTANT NOTES	Additional publications/papers/survey in a PDF format related to the patient’s condition
CONTACT	Patient’s contact mobile number

**Table 2:** List of the types of genetic testing requested in the genetic clinic.

Test Type	Test Name
Chromosomal testing	Chromosomal analysis (karyotyping) Chromosomal breakage Fragile X chromosomes
Biochemical tests	Metabolic screening test (urine or blood) Enzyme assay Serum amino acids
Molecular testing	FISH analysis (for microdeletion or any specific loci) A DNA Microarray Methylation status analysis Sequencing for a specific gene Whole exome sequencing (WES) Whole genome sequencing
Testing for blood disorders	Hb electrophoresis
Preimplantation testing	PGD for single gene disorder or for specific chromosomal abnormality

**Table 3:** Entities and attributes type.

Data Element	Description	Classification	Type	Role
**PATIENT/Entity**				
MRN	Patient’s Medical Record Number	Attribute	Identifier	Primary Key
**DEMOGRAPHICS/Entity**				
DEMOGRAPHICS_ID	Uniquely identify the entity	Attribute	Identifier	Primary Key
NAME	Patient’s name	Attribute	Descriptor	
PATIENT_GN	Patient’s Genetic Number	Attribute	Descriptor	
GENDER	Patient’s sex	Attribute	Descriptor	
DOB	Patient’s date of birth	Attribute	Descriptor	
NATIONALITY	Patient’s nationality	Attribute	Descriptor	
ADDRESS	Patient’s address	Attribute	Descriptor	
CONTACT	Patient’s mobile number	Attribute	Descriptor	
**FAMILY_HISTORY/Entity**				
FAMILY_HISTORY_ID	Uniquely identify the entity	Attribute	Identifier	Primary Key
INHERITANCE_PATTERN	Patient’s Inheritance Pattern	Attribute	Descriptor	
CONSANGUINITY	Parent’s consanguinity	Attribute	Descriptor	
MOTHER_AGE	Patient’s mother’s age	Attribute	Descriptor	
DNA_AVAILABILITY	Patient’s DNA test results	Attribute	Descriptor	
PEDIGREE_NAME	The filename for a chart tracing the inheritance of one or more traits through a family	Attribute	Descriptor	
PEDIGREE_TYPE	The file type	Attribute	Descriptor	
PEDIGREE_CONTENT	Data (content)	Attribute	Descriptor	
**PHYSICIAN/Entity**				
PHYSICIAN_ID	Uniquely identify the entity	Attribute	Identifier	Primary Key
PHYSICIAN _NAME	Physician’s name	Attribute	Descriptor	
CONTACT	Physician’s mobile number	Attribute	Descriptor	
**CONDITIONS/Entity**				
CONDITION_NAME	Patient’s condition’s name	Attribute	Identifier	Primary Key
**CLINICAL/Entity**				
CLINICAL_ID	Uniquely identify the entity	Attribute	Identifier	Primary Key
CLINICAL_DESCRIPTION	Phenotypes information of the patient which is related to the specific condition	Attribute	Descriptor	
**TEST/Entity**				
TEST_ID	Uniquely identify the entity	Attribute	Identifier	Primary Key
TEST_TYPE	The specific test type	Attribute	Descriptor	
**SAMPLE/Entity**				
SAMPLE_ID	Uniquely identify the entity	Attribute	Identifier	Primary Key
SAMPLE_TYPE_	Specify the sample type	Attribute	Descriptor	
**PLAN/Entity**				
PLAN_ID	Uniquely identify the entity	Attribute	Identifier	Primary Key
PLAN_TYPE	The name of the plan	Attribute	Descriptor	
**PHOTO/Entity**				
PHOTO_ID	Uniquely identify the entity	Attribute	Identifier	Primary Key
PHOTO _NAME	The name of the photo	Attribute	Descriptor	
PHOTO _TYPE	The type of the photo	Attribute	Descriptor	
PHOTO _CONTENT	Patient’s photo (data)	Attribute	Descriptor	
**DOCUMENT/Entity**				
DOCUMENT _ID	Uniquely identify the entity	Attribute	Identifier	Primary Key
DOC_NAME	File name	Attribute	Descriptor	
DOC_TYPE	File Type	Attribute	Descriptor	
DOC_CONTENT	Any important notes, reports, and literature in PDF format (data)	Attribute	Descriptor	
DOC_DESCRIPTION	Text to describe the document type	Attribute	Descriptor	

**Table 4:** The multilevel service design approach for the development of the G3DMS.

MSD Steps	The G3DMS Application
**Step 1: Study the system user’s experience**	Case Study: the PACER-HD Focus group, brainstorming method for discussing system requirements (process and data)
**Step 2: Design the service concept**	The strategy phase of the system design lifecycle Display (the basic ERD and the DFD level zero)
**Step 3: Design the service system**	The analysis phase of the system design lifecycle Display (the global schema, and the DFD level one)
**Step 4: Design the service encounter**	The design, the build, the documentation, the transition, and production phases of the system design lifecycle Display (the logical schema, site map, the wireframe diagram, user manual)

**Table 5:** The informatics evaluation framework for the G3DMS.

Stage of System Development	Level of System Evaluation	The G3DMS Evaluation Method
**Stage 1: Specification and Needs Requirement**	Evaluate specifications	A brainstorming method is carried out with a focus group from the PACER-HD, including the head of the organization, four physicians, and the author. The purpose of this method is to evaluate and define the problem and acquire the requirements (process requirements and data requirements).
**Stage 2: Component Development (the database and the user interface)**	Evaluate in the lab	Unit testing (database and the user interface) performed in the development environment (the XAMPP platform) using the MySQL module for database testing through phpMyAdmin and the application interface in the Apache server module through the localhost at port: 8080. The aim of these tests is to evaluate the programming code functionality of the user interface and query performance in the database.
**Stage 3: Combine Components**		The whole system interaction (user-interface and the database) is tested in the same environment. The goal of these tests is to evaluate the interaction and response between the front-end user interface and the back-end database.
**Stage 4: Integration of Components into System**	Evaluate in the field	The completed version of the system was tested in the local environment (the author’s laptop) at the PACER-HD center by two physicians under the author’s observation, and feedback notes were taken during the trial session. The purpose of this evaluation is to capture feedback and observe the user interaction with the system in term of difficulties and ease of use.
	Evaluate validity	Following the system deployment on the Internet, a link to the system is provided to the designated physician from PACER-HD with a username and password. A validation test checklist is also sent by email to help with the assessment as well as the user manual which can explain the functionality of all the interface pages and all the messages that may appear during user interaction with the system. The aim of this evaluation is to allow the system users to test the usability of the system without the influence of the author.
**Stage 5: Routine Use of a System**	Evaluate efficacy	The system is at its early stage as the PACER-HD management planning for transforming their legacy data to the G3DMS. After using the system for a period of three months, the FITT framework will be used to evaluate the user, system, and process interaction using the appropriate method for data collection (either quantitative, interviews, or focus group) to evaluate the system. The purpose of this evaluation is to determine the effectiveness of the system in the care process and for research studies.

The evaluation framework. Presented by (Kaufman et al., 2006) [31], adopted from (Stead et al., 1994) [32].

**Table 6:** The action plan evaluation.

Goal			
To Design and Implement a Data Management System for the Diagnosis of Genetic Disorders			
**Description**	**Team Members**		**Role**
**Planning team**	Mrs Halima Samra, PhD candidate, La Trobe University, Melbourne, Australia		System developer
	Prof. Ben Soh, Associate Professor, La Trobe University, Melbourne, Australia		Supervisor
	Dr Alice Li, Senior Lecturer, La Trobe University, Melbourne, Australia		Co-Supervisor
	Chairman of Dept of Genetic Medicine, Faculty of Medicine and Director of PACER-HD—Jeddah-KSA		Higher Management
	A researcher at the PACER-HD		Super User
	A research Assistant and Lab Coordinator at the PACER-HD		System user
	Three physicians who are researchers at the PACER-HD		System user
**Description**	**Diagnosis Workflow**	**Outcomes**	**For Research Study Purpose**
**The current system challenges in PACER-HD**	All the process was done manually, and patient data were stored in a paper-based format (test results, reports, photos, and research papers).	An unwieldy way of reading through all paper-based documents and drawing conclusions for the purpose of patient diagnosis. It is almost impossible to compare data of multiple patients in order to confirm diagnosis.	All data collected manually then entered to Excel files for analysis. However, it is difficult to answer complex research questions.
**The G3DMS**	Support standardized data collection using electronic forms for structured data collection, storage, and processing, as well as allowing PDF files and all types of images to be uploaded.	Easy retrieval of patient data and access to all data and files electronically with the possibility of reviewing them with the results of other patients.	Allow uploading old data from Excel files. Provide query interface pages to support simple, advanced, and complex research questions, in addition to export data from the database in Excel and JSON format.
**Action plan for the design and development of the G3DMS**			
**Task Description**	**Member**	**Resources**	**Evaluation**
**Problem definition, requirements and system specification**	The system developer, higher management, super users, and other users from research groups	Members meeting in a focus group discussion	The task accomplished its goal and was completed on time without delay
**Prepare conceptual frameworks**	The system developer	Present the model to the Super User for confirmation of data element and diagnosis process flow	The task outcome was satisfactory with minor amendments on data elements to suit the patient-centered design approach
**Prepare the logical frameworks**	The system developer	Present the model to the Super User for confirmation of data element and diagnosis process flow	The resulted blueprint was clearly understood by the user
**Programming and coding for both database and user interface**	The system developer	Development environment in the developer personal computer	Iterative testing until developer satisfaction
**Documentation**	The system developer	Step-by-step action scenarios with screenshots converted to a PDF file	This task resulted in a useful user guide
**Implementation and system deployment**	The system developer	Hiring a virtual private server (VPS); Super User and other users for testing the system; sample data for testing	Satisfactory outcomes, no errors, appropriate messages, accurate results
**Supervision and review**	Supervisor and Co-Supervisor	Each stage documentation and coding	Approval of submitted work for each phase
